# Emerging Domains for Measuring Health Care Delivery With Electronic Health Record Metadata

**DOI:** 10.2196/64721

**Published:** 2025-03-06

**Authors:** Daniel Tawfik, Adam Rule, Aram Alexanian, Dori Cross, A Jay Holmgren, Sunny S Lou, Eugenia McPeek Hinz, Christian Rose, Ratnalekha V N Viswanadham, Rebecca G Mishuris, Jorge M Rodríguez-Fernández, Eric W Ford, Sarah T Florig, Christine A Sinsky, Nate C Apathy

**Affiliations:** 1 Department of Pediatrics Stanford University School of Medicine Palo Alto, CA United States; 2 Information School University of Wisconsin-Madison Madison, WI United States; 3 Department of Family Medicine Novant Health Winston-Salem, NC United States; 4 Division of Health Policy and Management University of Minnesota School of Public Health Minneapolis, MN United States; 5 School of Medicine University of California San Francisco San Francisco, CA United States; 6 Department of Anesthesiology Washington University School of Medicine St Louis, MO United States; 7 Department of General Internal Medicine Duke University School of Medicine Durham, NC United States; 8 Department of Emergency Medicine Stanford University School of Medicine Palo Alto, CA United States; 9 Department of Population Health New York University Grossman School of Medicine New York, NY United States; 10 Division of General Internal Medicine Brigham and Women's Hospital Boston, MA United States; 11 Department of Neurology School of Medicine University of Texas Medical Branch Galveston, TX United States; 12 Department of Health Policy and Organization School of Public Health University of Alabama Birmingham, AL United States; 13 Department of Pulmonary, Allergy, and Critical Care Medicine Oregon Health & Science University Portland, OR United States; 14 American Medical Association Chicago, IL United States; 15 Department of Health Policy & Management University of Maryland School of Public Health College Park, MD United States

**Keywords:** metadata, health services research, audit logs, event logs, electronic health record data, health care delivery, patient care, healthcare teams, clinician-patient relationship, cognitive environment

## Abstract

This article aims to introduce emerging measurement domains made feasible through the electronic health record (EHR) use metadata, to inform the changing landscape of health care delivery. We reviewed emerging domains in which EHR metadata may be used to measure health care delivery, outlining a framework for evaluating measures based on desirability, feasibility, and viability. We argue that EHR use metadata may be leveraged to develop and operationalize novel measures in the domains of team structure and dynamics, workflows, and cognitive environment to provide a clearer understanding of modern health care delivery. Examples of measures feasible using metadata include quantification of teamwork and collaboration, patient continuity measures, workflow conformity measures, and attention switching. By enabling measures that can be used to inform the next generation of health care delivery, EHR metadata may be used to improve the quality of patient care and support clinician well-being. Careful attention is needed to ensure that these measures are desirable, feasible, and viable.

## Introduction

Since the widespread adoption of electronic health records (EHRs) following the enactment of the Health Information Technology for Economic and Clinical Health (HITECH) Act, clinical care is increasingly mediated by health information technology (HIT) [1]. The digitization of health care has enabled not only the capture of more clinical data for use in clinical research [2], but also metadata about how those data are produced and used, giving a window into the process of health care delivery [3-7].

Established health care metrics focus largely on the patient process and outcome measurements (eg, health maintenance screening and preventable admissions) or outcomes for individual health care workers (eg, turnover and professional burnout). While these measures reflect important outcomes of a complex health care system, they do not reliably indicate upstream aspects and contextual factors of health care delivery, which may be leveraged to improve patient and clinician outcomes. EHR use metadata (eg, audit logs, orders metadata, documentation and communication metadata, and patient encounters metadata) contain valuable details on the complex system in which health care is delivered, with early insights primarily focused on discrete action or time-based measures [8-15].

This paper describes 3 examples of emerging domains of measurement through EHR use metadata, identified through a series of workshops facilitated by the Measures Workgroup of the National Research Network for EHR Audit Log Data [16]. These domains, health care teams, workflows, and cognitive environments, may provide a clearer understanding of modern health care delivery. We expect the value of these measures to outweigh the resources required to develop them, informing the next generation of health care delivery that improves both the quality of patient care and supports clinician well-being.

## Data and Data Sources

Traditional sources of health care delivery data (eg, time and motion studies, surveys, or administrative claims) [17,18] often require high resource expenditure to capture accurately and in detail. In contrast, continuously collected EHR use metadata can facilitate widespread measurement of several domains of health care delivery [4,7,13], providing valuable data that otherwise would require direct observations that are virtually impossible to collect at scale or for extended durations [19].

At a more granular level, EHR audit logs and other event logs offer a detailed account of actions that took place within the EHR at what time and by whom [20], enabling exploration of the “path” that a clinician or team took in the EHR to complete that task. In turn, these details can be used to delineate certain workflows or team structures contributing to task efficiency and clinical outcomes, which may be the result of vendor-derived or investigator-derived measures [21]. Such approaches have been described for a variety of EHR vendors, clinical scenarios [12], methodologies [22], and health professions [23].

Although metadata are not identical across EHR vendors, and institutions often have differing customizations and third-party modules, commonalities among metadata structures make it feasible to normalize measures across institutions with sufficient considerations. In addition, recent studies from 2 of the largest EHR vendors suggest that clinician variation far exceeds organizational variation [24,25], indicating that metadata-derived EHR use measures are more reflective of individual workflows than organizational configurations. We posit here that these new horizons of measurement warrant dedicated development and prioritization of the key clinical and operational questions for which EHR use metadata can be brought to bear.

## Principles of Domain Measurement

**Figure 1:**
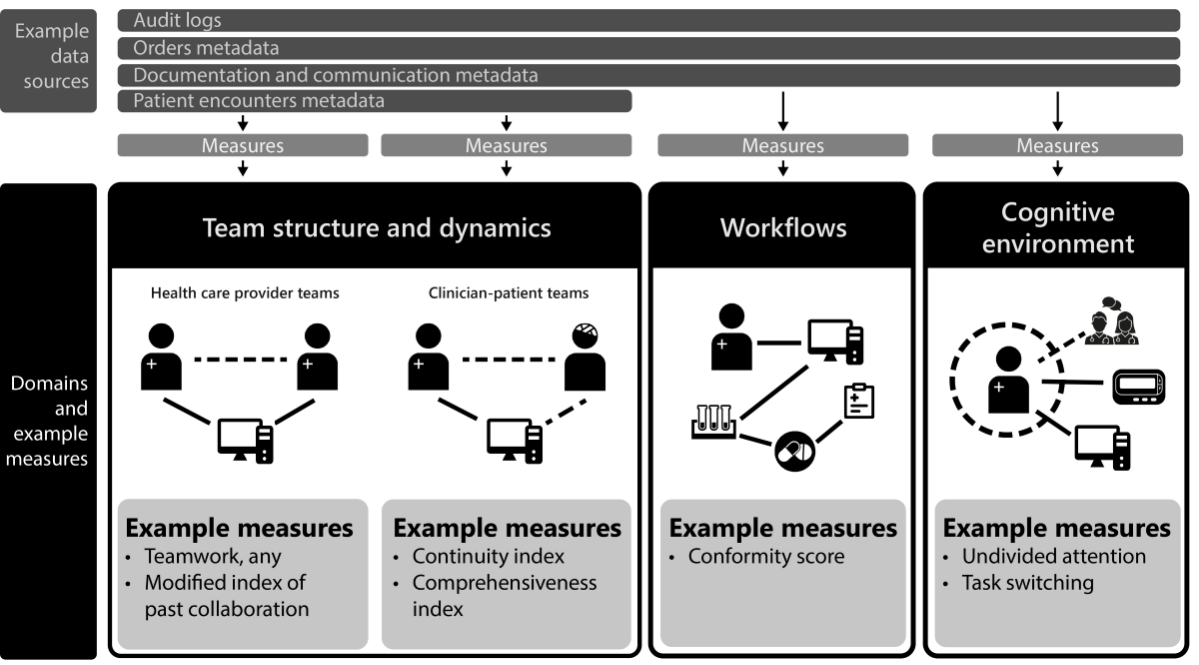
Emerging domains of measurement: team structure and dynamics, workflows, and cognitive environment, with example measures for each.

Innovative ideas need to be feasible, viable, and desirable to be successful [26]. While originally developed for commercial products, this framework can also guide EHR measure development. First, EHR use metadata to enable new measures of health care delivery that are technically feasible but also require proof that such measures are valid and reliable. Second, analyzing EHR metadata takes less time and money than many other methods of observing health care delivery, but measurement schemes based on EHR metadata still need to prove their viability (providing more value than the effort required to produce them) by generating insights that tangibly improve care [27]. Finally, measures need to be desirable, aligning with human values and providing insight into phenomena people care about. Desirable measures may answer research questions, contribute to more efficient and reliable single-center cross-sectional or longitudinal operational and quality reporting [28], or facilitate multi-institutional comparisons. This perspective demonstrates that, in health care delivery, these phenomena include not only how people spend their time but also how they interact (team structure and dynamics), organize their work (workflows), and direct their attention (cognitive environment; Figure 1) [9,21,29-33].

Making decisions based on social measures is fraught with risk, with a natural tendency to focus on what is easiest to measure (feasible) rather than what is most useful (viable) or meaningful (desirable) as an indicator of high-quality processes or outcomes [34]. Yet, most measures are only rough proxies for more complex phenomena, and improving the measure (eg, patient satisfaction scores) may not improve the phenomenon of interest (eg, patient satisfaction) [35]. Furthermore, poor selection of a proxy can promote bias (eg, using predicted health care costs as a proxy for clinical risk underestimates the needs of those with less access to care [36]), and measures used for social decision-making are more likely to be gamed [37].

These risks do not require that we avoid measuring health care delivery. As statistician George Box observed, “Essentially, all models are wrong, but some are useful” [38]. Developing useful measures while acknowledging the ways in which they are wrong requires careful attention to the social aspects of what cannot be easily measured and the unintended consequences of putting such measures into practice [39]. Here we describe 3 domains of health care delivery where EHR use metadata measures may be feasible, viable, and desirable, though not without risk.

## Measuring Team Structure and Dynamics

Most patient care is delivered through teams of nurses, medical assistants, technicians, pharmacists, physicians, and many other staff members all of whom are integral to care delivery and outcomes. Fortunately, a byproduct of EHR auditing requirements means that EHR use metadata record details on both the clinicians and patients for whom they perform actions, making it feasible to reconstruct team structures and dynamics. This can be viewed as (1) clinician-centric models of health care provider teams, and (2) patient-centric models of clinicians involved in their care (synchronously and not), recognizing that each feeds back on the other through complex relationships. Identifying these team structures relies on inference based on shared patient access, physical locations of EHR access, temporal proximity, or direct communication records [21,40].

## Health Care Provider Teams

EHR uses metadata in relation to defined team structures to allow investigation into patterns of how health care teams interact with each other. The “strength” of team structure can then be weighted (eg, through repeat interactions) to quantify team familiarity that might promote shared mental models that are essential for effective teamwork.

Researchers have the opportunity to consider the timing, intensity, and content of team-based interactions as proxies for effective teamwork. This requires a robust understanding of how EHR-based teamwork takes place in a broader framework of in-person and HIT-mediated collaboration, allowing for variance in the phenotypes of team collaboration that can reliably still be associated with provider satisfaction and patient care quality [41]. This work also requires the development of outcome measures more sensitive to the quality of team collaboration, such as care delays or therapy cycling. Valid and reliable understanding of the connections between team structure, processes, and outcomes is foundational to each measure’s viability and desirability, insofar as it supports health care managers in making operational decisions and designing institutional supports that enhance team collaboration and quality of care [42].

## Clinician-Patient Teams

The clinician-patient relationship is the cornerstone of health care provision, yet many essential measures of this relationship have been prohibitively difficult to measure at scale before the emergence of EHR use metadata. For example, continuity of care is associated with patient satisfaction and a host of improved outcomes [43,44], attributed to the trust and knowledge in the clinician-patient relationship that supports tailored evaluations, accurate diagnosis, customized treatment plans, and increased medication adherence. EHR use of metadata has made more nuanced measurements in this domain feasible because system-wide records of all clinician-patient interactions now enable quantifying repeated interactions with the same clinician at a more granular level than visits, procedures, or diagnoses. These records include asynchronous messages through patient portals and documentation of telephone encounters. With the addition of domain knowledge, these detailed metadata also enable assessments of the appropriateness of a patient’s encounters with primary care versus subspecialty care. These concepts of continuity of care [45-53] and comprehensiveness [31,54,55] are particularly viable and desirable as they may help physicians and operational leaders optimize care to achieve the quintuple aim [27,56] goals of quality, cost, satisfaction, patient and care team experience, and equity.

Through improved measures and stakeholder engagement, health care systems can work to optimize the relationship between clinicians and patients, to promote health care that is not only more effective and efficient but also more satisfying and rewarding for both clinicians and patients.

Health care team structure and dynamics are complex and not fully reflected within EHR use metadata, raising important limitations. The balance between HIT-facilitated and in-person interactions varies among settings, with important implications for the validity of extrapolating teamwork measures from EHR use metadata within a particular use case. In addition, value judgments on the appropriateness or quality of a particular team structure or clinician-patient interaction require additional clinical and system knowledge, much of which is not readily available within EHR data or metadata.

## Measuring Workflows

EHR audit logs and other event logs contain records of timestamped granular interactions with an EHR as part of larger clinical tasks (eg, refilling a prescription or ordering bloodwork), making feasible large-scale identification of common pathways for routine clinical practices [32]. These pathways may then be evaluated for relative time spent on clinical tasks, the complexity of action sequences, engagement with decision support, variation for particular workflows, specialties, providers, or combinations of these (eg, variation in outpatient ordering workflow among physicians within 1 clinic) [57].

To be viable and desirable, workflow measures must account for the tension between unnecessary and necessary deviations of behaviors from the norm [58]. Reducing unnecessary deviations can improve efficiency and patient safety through standardized best practices [59,60]. For example, heterogeneity in physician work with medical scribes may negatively impact physician efficiency [61]. While highly variable stroke care pathways may result in therapeutic delays [32]. Yet, enabling necessary deviations from the norm may promote tailored approaches to unique or complex scenarios, promoting clinician autonomy, professional satisfaction, and precision medicine.

Key to this measurement domain is the risk of misclassifying an unhelpful workflow as “normative” or a necessary variation as “deviant,” highlighting the importance of establishing clinical expertise, stakeholder buy-in, and relationships to important outcomes in the development and deployment of any workflow measures. In addition, although efforts at automated task identification within audit log data have been made [22,62,63], the diversity of clinical workflows makes reliable task identification challenging. Furthermore, due to differences in actions and tracking methods available in particular EHR instances, such measures may be poorly generalizable outside of an institution or EHR vendor unless carefully designed to be context-agnostic and may require recalibration over time with new technologies (eg, large language models and generative artificial intelligence), clinical approaches, or software functionality.

## Measuring Cognitive Environment

Clinical work is cognitively complex; clinicians must synthesize ever increasing amounts of information to make diagnosis and treatment decisions amidst competing demands for their attention. Cognitive overload, which has been linked to errors and burnout, can occur easily in such environments [64,65]. Because clinical work is largely mediated by the EHR [66], EHR use metadata offers opportunities to measure clinician cognitive load at scale without laborious data collection. These measurements are critical for understanding how the clinical cognitive environment can be improved.

Cognitive load includes an intrinsic component related to the complexity of a particular task (eg, patient complexity), and an extrinsic component related to the work environment (eg, interruptions, poor EHR, or workplace design [67]). Both components may be feasible to measure using EHR use metadata. For example, intrinsic cognitive load can be approximated using audit log–derived measures of patient load [11,68], EHR time [69], and encounter or task complexity [70]. Extrinsic cognitive load is more challenging to measure; while some interruptions like secure messaging use can be observed directly within domain-specific or third-party event logs [71], many occur outside of HIT. Instead, efforts have focused on capturing task fragmentation [72,73], such as attention switching [74] or undivided attention [9,33,65,67,74-83].

While this domain has considerable promises, there are several barriers to progress. Few of the audit log-derived cognitive load measures have been validated using established instruments from the cognitive science literature (eg, surveys like the NASA-TLX [National Aeronautics and Space Administration–Task Load Index] or physiological measurements like pupillometry) [84]. In addition, difficulties with the related concept of task identification limit the ability to subsequently measure task fragmentation, with further validation of task identification needed before task fragmentation can be reliably measured.

## Conclusion

EHR uses metadata to provide remarkable potential for measuring and improving health care delivery by making feasible previously inaccessible insights. By measuring team structure and dynamics using intersecting metadata on clinician EHR use, health care provider teams may be studied and optimized and clinician-patient teams can be improved to prioritize continuity of care and comprehensiveness where appropriate. Health care workflows may be better understood through better identification of tasks and measurement of paths taken to achieve them. Finally, proxies of cognitive load may be measured, to inform interventions to reduce overload and the resultant risks for burnout and medical errors. Each of these measurement domains will require further tuning to fully realize their promise of improving health care delivery through sustainably scalable measurement.

## Abbreviations

EHR: electronic health record
HITECH: Health Information Technology for Economic and Clinical Health
HIT: health information technology
NASA-TLX: National Aeronautics and Space Administration–Task Load Index
